# Adapting to the Needs of the Public Health Workforce: An Integrated Case-Based Training Program

**DOI:** 10.3389/fpubh.2016.00221

**Published:** 2016-10-14

**Authors:** Shannon L. Sibbald, Mark Speechley, Amardeep Thind

**Affiliations:** ^1^Family Medicine, Western University, London, ON, Canada; ^2^Health Sciences, Western University, London, ON, Canada; ^3^Schulich Interfaculty Program in Public Health, Western University, London, ON, Canada; ^4^Epidemiology and Biostatistics, Western University, London, ON, Canada

**Keywords:** interprofessional education, public health education, pedagogy, case-based questioning, training

## Abstract

The goal of any public health education at the Masters level is to transmit knowledge and skills to meet current and future public health challenges. We suggest an innovative multi-modal approach to public health education using a case-based pedagogy combined with competency-based curriculum and a team-based approach to foster truly experiential learning. We describe each pedagogical approach in connection to the relevance of optimal methods for training public health professionals. Western University’s Schulich Interfaculty Masters of Public Health (MPH) program (ON, Canada) provides a unique interprofessional education through case-based learning and competency-based curriculum. This Masters program has attracted applicants from around the world to learn in a supportive interprofessional environment and to foster them as they become learners and leaders in public health changes. To our knowledge, we are the first condensed MPH program using integrated case-based pedagogy as our main pedagogical approach.

## Background and Rationale

Health professional education is in a constant state of change in order to meet the demands of a very complex work force. Training of health professionals has shifted focus to be more about skills and competencies (i.e., “what can you do”), rather than about specific information (i.e., “what do you know”). The impact of changes in training goals has dictated the need for transformations in how programs conceive, organize, and provide education to meet current and future health challenges.

Public health is an interdisciplinary field that requires health professionals working in it to be proficient in a myriad of skills and competencies not only for their own profession but also of other professions with whom they work. Postgraduate programs specific to public health provide the required establishment for training a competent workforce and avoid gaps in knowledge and skills. There exists a growing need for high quality postgraduate public health educational programs, as well as rigorous continuing education specific to public health.

The Council for Education in Public Health (CEPH) defines public health as, “enhancing health in human populations, through organized community effort” (1). CEPH views public health as a distinct area of practice, like dentistry or nursing. Over the past 25 years, public health education in Canada has expanded rapidly, with the number of postgraduate public health programs tripling since the 1990s (2). The public health workforce involves at least 25 unique regulated professionals, who must be highly competent in order to carry visions of system change into reality. However, training programs lacking a public health practice orientation are still relied on; many public health workers are trained in disciplines other than public health, using discipline-specific competencies that do not always map onto public health-specific competencies.

Since the 1970s, emphasis upon collaboration among health-care professions has been increasing, and in order to facilitate this way of working, interprofessional education (IPE) has been introduced (3). IPE has been defined as, “an intervention where members of more than one health or social profession, or both, learn interactively together for the explicit purpose of improving interprofessional collaboration or the health/well-being of patients/clients or both” (4). While the effectiveness of IPE on learning outcomes is not entirely clear (4), the importance of IPE has been recognized across the health system – for example, in the Ontario Health Action Plan (5) and in Ontario’s Public Health Sector Strategic Plan (6). The First Ministers’ Accord in 2003 included a health human resources strategy to promote IPE, thereby advancing collaborative care (7). In 2004, the First Ministers renewed their commitment to supporting IPE by adding a Canadian Health Human Resources Strategy (8). The interest and focus on IPE have been evident in the changing curriculum in health professional education. Given the recent changes in program delivery, coupled with rising expectations of the public health workforce, novel approaches in public health education are required.

In an effort to expand and innovate in public health education, our program faculty has carefully considered both the context of learning as well as the transmission of information to meet current and future public health challenges. Western University, located in Ontario, Canada is home to 30,000 students and 12 faculties and schools. Western’s Schulich Interfaculty Program in Public Health is housed within the Schulich School of Medicine and Dentistry. The program trains a maximum of 60 students in an intensive 1-year course-based professional Masters of Public Health (MPH) program. This innovative case-based program has attracted applications from around the globe. Our case-based, competency-based program was built for the increasingly interdependent demands of an ever-changing global health system.

Western’s goal was to create a unique and focused program grounded in transformative learning. The goal of this paper is to present an overview of current practices in public health education at the Masters level. We will explore how both the case-based approach and competency-based education models prepare students for the complexities of real-world decision-making. We believe our approach is both an innovative and essential approach to teaching in the complex area of public health.

## Learning Objectives

Public health by definition is interdisciplinary and, as such, the objectives of our MPH program are diverse. Our program not only imparts solid technical skills and promotes critical thinking through competency-based curriculum but also embolden our students to be conscientious interdisciplinary professionals through an IPE lens. Our students take 49.5 credits in a condensed 1-year program, resulting in a very intensive learning experience. Each course has its own learning objectives, which align with our program competencies (discussed below). Our program’s mission is “to produce transformative knowledge, professionals and leaders that work to create healthy and sustainable communities both locally and globally. To this end, we foster professional education, public outreach and scholarship to support practitioners, leaders and change agents in moving societies toward more sustainably healthy futures”.

## Pedagogical Frameworks

While there is no one best, agreed-upon, approach to interprofessional teaching, research has shown specific pedagogic approaches to be better suited for interprofessional learning. These include problem-based learning (PBL) (9–12), small group and seminar discussions (12–15), role play and simulated ward experiences (15, 16), workshops with problem-based multidisciplinary case studies, team building sessions, student placements where shadowing can occur (17), and collaborative practice across different professions (18).

Principles of effective instruction emphasize incorporating a thoughtful collaboration among students as a technique to increase levels of engagement (19). We draw on three distinct and inter-related pedagogical approaches that allow for a high level of engagement and a more comprehensive learning experience:

(1) Case-based pedagogy
(2) Team-based learning
(3) Competency-focused curriculum

We have created a program whereby each component builds on and off the others and is inter-related and iterative. We believe our approach to education provides our students with the necessary skills and tools to succeed in the fast paced, dynamic, and complex work environment of public health.

### Case Based

The case method of learning, which we have adapted from business management education, requires students to take an active role in their learning. This learning-by-doing involves the exploration of complex and often ill-defined real-world public health issues. Students simulate the experience faced by actual decision-makers using imperfect information in a swiftly changing environment. Cases are our main educational material. Students are often provided with supplementary or additional readings (journal articles, text book chapters) as well; however, one of our internal outcome measures is that 60% of course teaching is case-based/experiential learning (see Table 1 for a description of the differences between experiential and lecture-based learning).

**Table 1:** **Contrasting extremes: lecture-based teaching vs. experiential learning**.

	Lecture based	Experiential learning
Terms and labels	Lecture method Didactic (lecturing) “Student”	Discussion teaching; active learning; case method learning; problem-based learning; advanced seminar; simulations “Learner”
**Characteristic**		
View of learning	Learning-as-product; can be precisely measured Learning ends with last lecture Learning is information	Learning-as-process: cannot be precisely measured Learning is life-long skill Learning is experience
**Learning style**	**Passive**	**Active**
Student preparedness, attendance and participation	Varies; can be entirely optional	Preparation, attendance, and participation essential to learning
Responsibilities	Professor has major responsibility for teaching, which is transferring information	Learners have major responsibility for learning, both for themselves and contributing to the learning of their colleagues, in both individual and group settings
Optimized for	Small details Memorization Un-integrated bits of information Facts: “right answers” A “canon” of core concepts Short-term recognition Knowing “about” Certainty	Large, broad concepts Application Synthesized, integrated knowledge Ideas: “alternative approaches” Skills and competencies Long-term understanding Knowing “how to” Uncertainty
Rate of “information transfer”	High: many “facts per hour”	Low – few “facts per hour”
Faculty resources	Fewer faculty resources required	Substantial faculty resources required
Role of theory	Theory as end in itself	Theory informs practice; practice informs theory
Role of professor	Professor is most important teacher Professor is “expert instructor” Professor teaches students	Learners become their own best professors Professor is “expert facilitator” Professor learns from students
	**Lecture based**	**Experiential learning**
Time allotted for questions and discussion	Varies from none to quite interactive; often spontaneous	Extensive, designed into each session
Visual aids	Slides prepared by professor	Words and diagrams drawn on board by professor and/or learners
Adaptability to emerging “news”	Less flexible/structured (because lecture topics and slides are pre-set)	Flexible/adaptable (i.e., can be a story from that morning’s news)
Order in which concepts are covered in a particular session	Often, largely predictable	Sometimes, largely unpredictable
When learning ends	Right around the final exam, when forgetting begins	Learning never ends if one has a learning need and the tools to locate knowledge

*Originally presented in Ref. (22)*.

There is no consensus on the definition of case-based learning (CBL). One definition includes viewing CBL in terms of its goal: “to prepare students for clinical practice, through the use of authentic clinical cases” (20). The method itself provides learners with substantive and procedural information that is essential to an analysis of a specific situation. It guides a reader to frame alternative action programs and considers the complexity and ambiguity of the practical world (21). By introducing a complex, ambiguous, real-world scenario, students must think and make decisions. In the case method, students have ownership of the discussion and are responsible for their learning. Authors of the present paper have suggested the following definition for public health case: “A real-world situation that promotes independent thinking as well as group discussion which ultimately allows the learner an opportunity to explore complex public health issues and apply theory to practice by analyzing, integrating and synthesizing knowledge” (p. 5) (22).

Case method learning is a three-stage process that builds on each subsequent step. It starts with individual case preparation, followed by a small group discussion, concluding with a large group discussion. Many disciplines use variations of experiential learning, including medicine, law, business, education, and engineering, as it challenges students to confront real-world situations. Case-based teaching has been proven to be superior to conventional didactic teaching in developing students’ decision-making and critical thinking skills (23). A recent report on teaching public health in undergraduate medical education found lectures to be the most prevalent method of teaching, followed by small-group teaching, then PBL; only 1.2% (*n* = 3) of medical schools were actively using the case-based method (24). CBL is often compared with PBL. PBL provides, “focused, experiential learning organized around the investigation, explanation and resolution of meaningful problems” (25). In PBL, “problems” tend to be individual patients or perhaps families, whereas public health cases have a wider organizational or community orientation (26). See Box 1 for a description of the difference between CBL and PBL.

Box 1 **Case-based method vs. problem-based learning.**

Problem-based learning is, “any learning environment in which the problem drives the learning” (26) – in other words, the learner is faced with a situation and realizes that basic knowledge must be identified and learned in order to understand the problem. Case based method involves learning-by-doing and the exploration of complex and often ill-defined real-world public health issues. Cases can be an example of PBL, although some business cases are designed to integrate previously learned concepts (26). Several schools have implemented case-based teaching in clinical health, such as McMaster and Northern Ontario School of Medicine (22).

#### Curricular Innovation

There is a paucity of teaching cases in public health. Case-based pedagogy is predominantly composed of business cases, while existing health-related cases tend to be based on the US and, therefore, do not reflect the reality of Canadian health systems. Case repositories (e.g., Harvard Publishing, Ivey Publishing, European Case Clearinghouse, etc.) have few teaching cases that can be used in a health program. This created an opportunity for our program to develop *de novo* cases that would align with course and curriculum objectives. We include case writing as both a training session and a requirement of our students. Students write cases based on their practicum; the outcome of this process has been the publication of two casebooks featuring selected cases, and an accompanying “instructor guidance” document written by our faculty and students (casebooks available for free download: http://www.schulich.uwo.ca/publichealth/cases/index.html). Our goal is to create a searchable database of freely available public health cases on our website, for use by any program across the world. Our faculty have actively incorporated these student cases in their curriculum, and we often involve the students (now alumni) in co-teaching these cases, which is an excellent mechanism for keeping alumni linked and committed to the program.

### Team-Based Learning

A pedagogy that emphasizes collegiality is an essential piece of public health education, fostering free thought to ensure a complete and mutual learning experience (27). We uphold high standards of professionalism and teach our public health program with collegiality, through innovative team-based learning techniques. On a typical day, teams meet for approximately 3 h. Teams are assigned and remain a unit for the duration of the program; we believe that successful teams are able to perform well together, but also improve with time (28). A recent review holds that successful teams share several common attributes, including: commitment to team success and shared goals; interdependence; interpersonal skills (openness, honesty, trustworthiness, supportive, and respectful); open communication and positive feedback (which includes active listening); appropriate team composition (role clarity); and commitment to team processes, leadership, and accountability (29).

Our teams have five to six students; group sizes between three and six students are optimal for collaborative learning (30). Foundational studies about the effect of group size on performance (31–33) and more recent research (34) have shown team size to be related to internal team communication, team performance, and leader behavior. Teams work toward understanding the various roles of each team member as well as fostering an understanding of professional differences and similarities (35). Each team has a faculty advisor who can help strengthen the trust and communication of teams if needed and help with conflict resolution when necessary.

We conceive team-based learning as a collaborative process where our students work together, drawing from discipline-specific perspectives to address a mutual problem. We argue, as do others (36, 37), that diverse and heterogeneous teams are the norm (especially in healthcare), and working effectively in such teams can lead to improved problem solving and increased quality of care. Students are assigned to their learning team prior to arriving in our program with an effort to ensure diversity in disciplines and demographics (namely country of origin). This diverse distribution of team members has been labeled “network heterogeneity”; increases in network heterogeneity facilitate learning and creativity and are thought to increase the level of team performance (38).

### Competency-Focused Curriculum

Researchers have outlined competency outcomes specific to IPE, such as communication, conflict resolution, problem solving, coping with uncertainty and ambiguity, group reflection practices, willingness to work together, mutual respect, and openness to trust in competencies of self and others (18, 35). Researchers have noted, however, that requiring interprofessional and collaborative activities does not always translate into tailored instruction or a formal assessment of competencies. While physicians and nurses have specified competencies, required competencies in other health professions are typically less well defined (39). Public Health Agency of Canada (PHAC) has seven public health-specific competencies: public health sciences; assessment and analysis; policy and program planning, implementation and evaluation; partnerships, collaboration, advocacy; diversity; communication; and leadership (40). PHAC states: core competencies, “are not designed to stand alone, but rather to form a set of knowledge, skills, and attitudes practiced within the larger context of the values of public health” (40). CEPH has the following competencies: biostatistics; epidemiology; environmental health sciences; health services administration; and social and behavioral sciences (1). Each organization provides a different view of public health competencies, which makes it difficult to determine the key expected competencies as public health professionals enter the workforce.

The core competencies outlined by the PHAC, and the Council of Education for Public Health were used as a guide in developing the 19 core competencies for students in our MPH program. Our MPH Program covers 19 competencies: competencies 1–8 include, for example knowledge of health systems and critically appraising the literature; these reflect the core areas of public health. Competencies 9–19 reflect our Program’s concentration and include designing a program evaluation and formulating effective health communication. See Table 2 for a list of our competencies.

**Table 2:** **Schulich MPH Competencies**.

**Core competencies**
Demonstrate knowledge of the systems in which public health functions, including current public health challenges (*Health Services Administration*)
Recognize how the determinants of health (biological, social, cultural, economic, and physical) influence the health and well-being of specific population groups (*Social and Behavioral Sciences*)
Perform a community needs assessment taking into account the unique social, environmental, economic, historical, and cultural characteristics of the community (*Social and Behavioral Sciences*)
Establish observable relationships between the present level of environmental stresses and human health (*Environmental Health Sciences*)
Critically appraise the literature to understand patterns of health and ill health, establish causal associations, and recommend courses of actions (*Epidemiology and Biostatistics*)
Demonstrate a professional appreciation of the ethical, legal, and social issues in public health policy and practice (*Health Services Administration*)
Identify participatory relationships to foster community collaboration (*Social and Behavioral Sciences*)
Develop and implement a sustainable plan to address public health challenge(s) (*Health Services Administration*)
**Competencies for concentration**
Recognize and apply effective leadership practices in the public health context
Promote leadership development by incorporating learning from self-reflection into professional development and public health practice
Formulate, for Aboriginal and other communities at risk, culturally relevant and appropriate strategies when planning, implementing, adapting, and evaluating public health programs and policies
Discuss the legal framework of public health practice including legislative authority, rights, obligations and risks, at the federal, provincial, and municipal levels
Optimize organizational performance by applying systems thinking
Design appropriate program evaluations for public health interventions
Critically assess research designs that are appropriate for public health practice
Write a basic research proposal for application in public health practice

## Pedagogical Format

There are two unique features to the success of our pedagogy:

(1) our dynamic learning environment
(2) our focused faculty and staff

### Learning Environment

Our program is housed within the Western Centre for Public Health and Family Medicine. Our dedicated space for students includes a large teaching classroom and 10 smaller learning team rooms. The learning environment extends beyond the classroom into our community and across national and international borders. Through our “Brown Bag Seminar Series,” we bring the world into our classroom, allowing the students to hear from and interact and network with practitioners from the field (such as current and past Chief Public Health Officers of Canada, Medical Officers of Health from provincial health units, WHO officials, NGO, and community health organization personnel). Our students then “go into the field” through practicum and community-engaged learning, providing the opportunity for learners to synthesize and integrate knowledge gained from coursework (1). Our program incorporates culminating experiences into our entire curriculum through a year-long course (transforming public health). Throughout the course, we organize 3-day-long integrated workshops. Students also take field trips to national and international public health organizations and attend regional and national public health conferences. This exposes them to a diverse array of practitioners and decision-makers, and allows them to network and learn about latest advances in the field.

### Faculty and Staff

The faculty of the MPH Program reflect the interlinked curriculum that they teach with expertise in anthropology, biostatistics, epidemiology, environmental sciences, family medicine, “big data,” health economics, health policy and law, health promotion, health services and implementation research, and management. As a new program, we identified and recruited interested (and deeply committed) faculty from across the different schools and departments at Western to the MPH program. This dual (and in some cases, triple) departmental appointment structure facilitates an interdisciplinary cross-talk and collaboration that would otherwise not have existed.

Student learning is also supported through our devoted program staff. A highlight is dedicated and individualized career counseling for our students both during the program and after graduation.

### Results to Date

The success of our MPH program is showcased throughout our circular innovations and national and international attention. The MPH program welcomed the first cohort of students in Fall 2013; the third cohort will graduate in August 2016. Applications have nearly tripled in the 3 years since the program started. The uniqueness of the program has enticed future public health leaders from Canada and the world. The level of interest increased the likelihood of an increased application pool (41). Most evaluation of teaching and course effectiveness across Canadian schools is done by the students (24). We also use this same model, and we invest in regular, more informal, mechanisms of evaluation. Students are asked to submit both midterm and year-end evaluations to the program, which cover program-specific measurements. Each year, students provide an overwhelmingly positive evaluation of our program, with some legitimate critical comments that we use for quality improvement purposes.

Almost all our graduates have been employed or admitted to other degree programs after graduation (including some who receive offers before they graduate), and anecdotal evidence suggests that employers (and students) highly value the case and team method of learning.

As a program, we have undergone rigorous evaluations in order to be a credible candidate for CEPH accreditation. The decision will be announced in the fall months of 2016.

## Discussion

Western has taken the lead in designing a novel case-based MPH program. We would like to not only share our experiences of the case method of learning in public health but also learn from other schools and programs doing the same. We would like to catalyze new initiatives and develop new interdisciplinary collaborations among public health educators and professionals, and this paper is a step in that regard.

### Practical Implications and Lessons Learned

#### Instructional Skill Set

Teaching using cases requires a different instructional skill set. Faculty must guide and manage class discussions and ensure that the learning objectives are met through this approach. We have sponsored our faculty to attend case teaching workshops, and our faculty are rapidly gaining experience in this approach through their own experience.

#### Case-Based Pedagogy

We also observed a lack of consensus on which public health topics are best taught by using cases. While topics such as leadership or program evaluation easily lend themselves to the case-based approach, the purely technical foundations of topics such as biostatistics and epidemiology do not. Our experience is that, although we can aim for 60% of our curriculum being taught using cases, there will, of course, exist wide variation between courses.

#### Team-Based Learning

Students (and employers) acknowledge the benefit of working in teams after graduation. However, incorporating this pillar of public health into an intensive 1-year curriculum can be challenging due to resource and time constraints, and each year there are teams that struggle. Faculty advisors invest time and energy guiding teams into developing strategies for working together. Despite the challenges, feedback from well-functioning teams indicates that this has been an excellent mechanism for building and fostering cross-cultural learning and understanding.

### Next Steps

As the MPH program grows, we are focusing our efforts on promoting the writing of public health cases, by reaching out to other schools/programs, public health practitioners, and researchers. Simultaneously, more thought has to be given to understanding what the appropriate teaching skills are for case method learning. How do we foster them? What effective facilitation skills are required? How do we use cases to sequentially present the mounting complexity of the core concepts of public health as the students move forward in their classes?

Last, but certainly not least, we need to build an evidence base to demonstrate the effectiveness of this format. What are the ideal methods of student and program evaluation of this format? The paradigm of transformative learning is underpinned by the sequential progression of the student from acquiring knowledge, to mastering the skills, and, finally, embodying the values of public health. How can we assess each step of this progression in a case-based education format?

## Conclusion

We believe our program is a success story. We have navigated the muddy and sometimes-torrential waters of public health education and come up with a unique and innovative approach that is producing graduates who go on to improved careers. Our global focus has inculcated a learning environment rich in experience and challenges. Our CBL team approach ensures our curriculum is relevant, current, and engaging. Case method learning is aligned with competency-driven instruction. Our competencies hold our students (and faculty) to a high standard, ensuring they will thrive in the complex challenges of public health. Ensuring high quality competency-based IPE leads to improved health-care delivery (42). We have more work to do; while this approach has been shown to be effective, it is a lot of work for faculty (43). We have plans to more formally evaluate our program and to share those results widely. Our casebook is, and will continue to be, available to all who wish to use it – the ethos behind the program is similar to that of public health: it affects all of us, so all should be able to gain.

## Author Contributions

All authors contributed to the ideas and conceptualization of the manuscript. SS drafted the first version of the manuscript. All authors edited and approved the final manuscript.

## Conflict of Interest Statement

The authors declare that the research was conducted in the absence of any commercial or financial relationships that could be construed as a potential conflict of interest.
